# Cerebral hyperperfusion syndrome after endovascular reperfusion therapy for medium vessel occlusion: A case report

**DOI:** 10.1016/j.radcr.2024.01.087

**Published:** 2024-02-20

**Authors:** Hideki Endo, Kohei Ishikawa, Ryota Nomura, Daishi Yamaguchi, Koichiro Shindo, Koji Oka, Hirohiko Nakamura

**Affiliations:** a Department of Neurosurgery, Nakamura Memorial South Hospital, 2-2, Kawazoe, Minami-ku, Sapporo, Hokkaido 005-8555, Japan; b Department of Neurosurgery, Nakamura Memorial Hospital, South 1, West 14, Chuo-ku, Sapporo, Hokkaido 060-8570, Japan

**Keywords:** Acute ischemic stroke, Anterior cerebral artery, Arterial spin labeling, Cerebral hyperperfusion syndrome, Endovascular treatment, Medium vessel occlusion

## Abstract

Cerebral hyperperfusion syndrome is a rare but serious complication after revascularization procedures for cerebrovascular diseases. Cerebral hyperperfusion syndrome can develop after treatment of acute ischemic stroke, including intravenous thrombolysis and endovascular treatment of large vessel occlusion. However, to the best of our knowledge, there are no previous reports describing cerebral hyperperfusion syndrome after endovascular treatment of medium vessel occlusion (eg, anterior cerebral artery A2/3 segment). We report a case of cerebral hyperperfusion syndrome after endovascular reperfusion therapy for medium vessel occlusion. A 70-year-old woman with a history of hypertension and dyslipidemia was transferred by ambulance to our hospital because of immobility and slurred speech. She had mild right lower extremity paralysis, and her symptoms appeared improved compared with onset. She was diagnosed with cerebral infarction in the left frontal lobe. After hospitalization, her neurological symptoms worsened and she was referred to our department. We performed endovascular reperfusion therapy for left anterior cerebral artery A2 occlusion. Recanalization was achieved with residual stenosis. Despite the lack of complications associated with the procedure, the patient had prolonged disorientation, severe hemiplegia, and aphasia. Arterial spin labeling demonstrated hyperperfusion in the left anterior cerebral artery area. The symptoms gradually improved under strict blood pressure control. This report provides evidence that cerebral hyperperfusion syndrome can occur even after endovascular treatment for medium vessel occlusion. Arterial spin labeling was useful in detecting hyperperfusion.

## Introduction

Cerebral hyperperfusion syndrome (CHS) is a rare but serious complication after revascularization procedures for extracranial and intracranial cerebrovascular diseases [1]. CHS is well known after carotid endarterectomy and carotid artery stenting, but it can also develop after endovascular treatment (EVT) for acute ischemic stroke (AIS) due to large vessel occlusion (LVO) [1], [2], [3]. However, to the best of our knowledge, there are no reports of CHS after EVT for medium vessel occlusion (MeVO). MeVO, defined as occlusion of the middle cerebral artery M2/3 segment, anterior cerebral artery (ACA) A2/3 segment, and/or posterior cerebral artery P2/3 segment, has recently been recognized as an indication for EVT [4]. Herein, we present a case of CHS after EVT for acute ACA A2 occlusion, which is one of the MeVOs.

## Case report

**Fig. 1:**
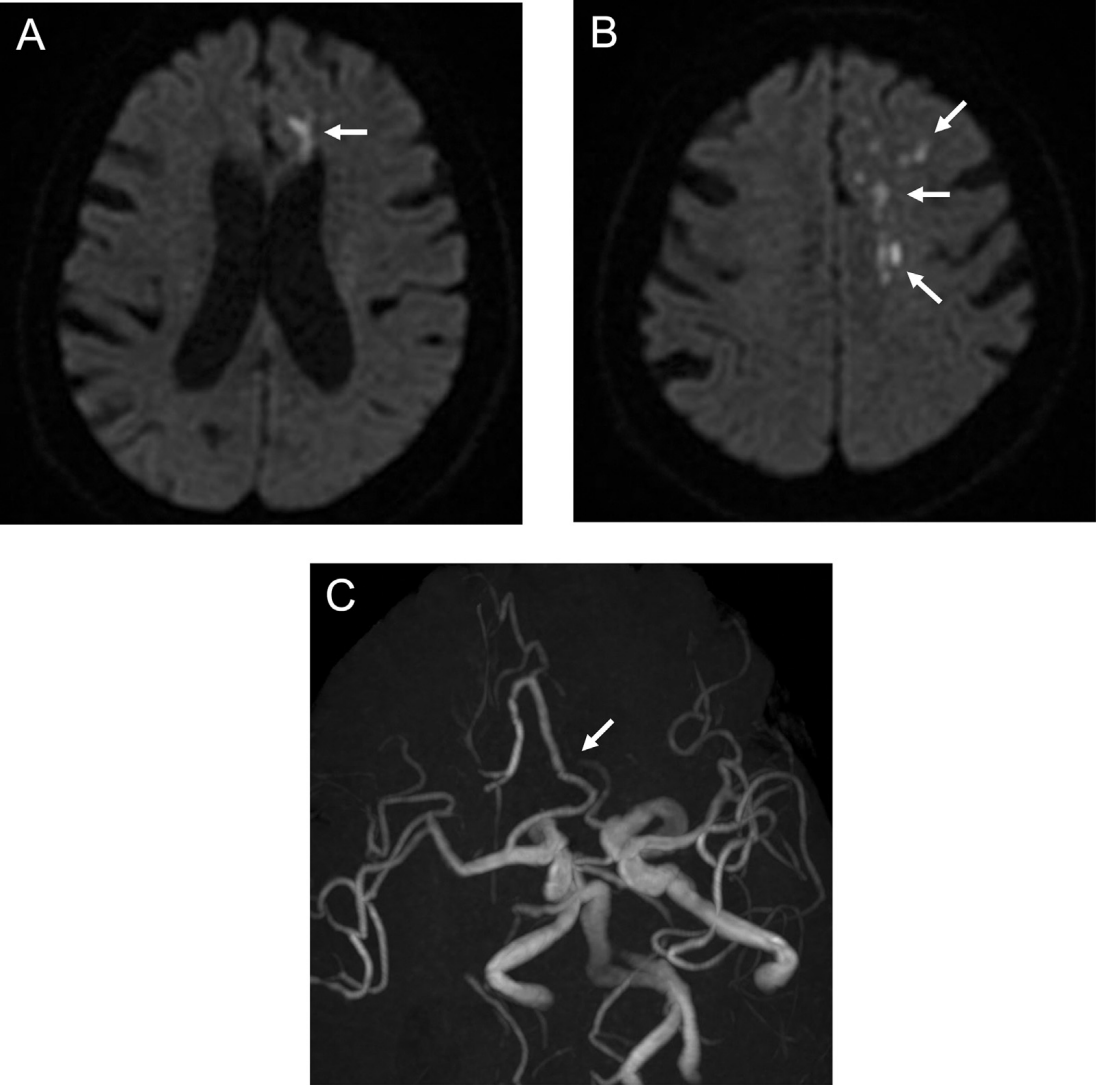
Preoperative magnetic resonance imaging and magnetic resonance angiography. Diffusion-weighted imaging showing multiple fresh infarcts (arrows) (A, B). Magnetic resonance angiography showing poor delineation of the left anterior cerebral artery, especially distal to the A2 segment (arrow) (C).

**Fig. 2:**
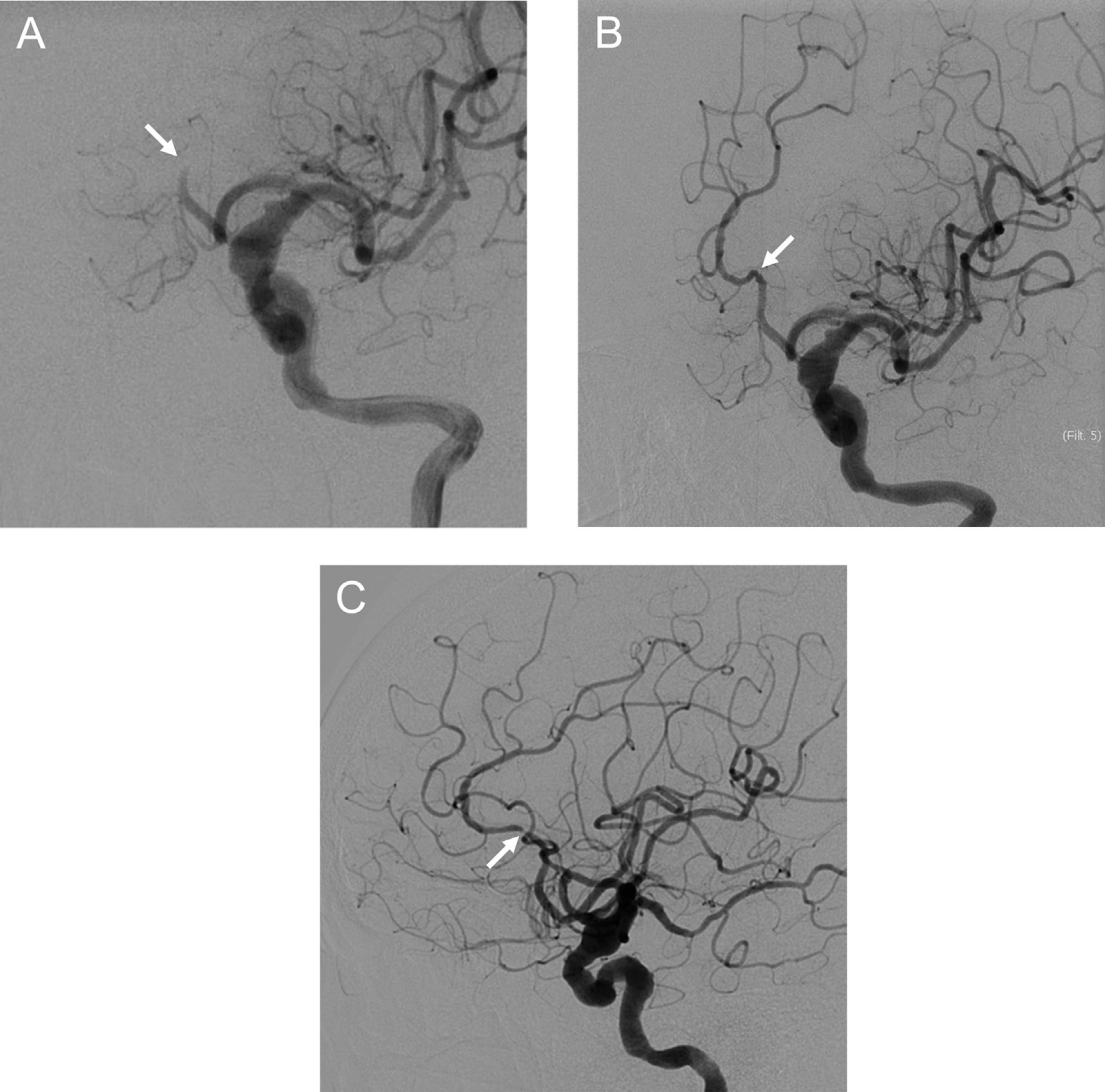
Angiographic findings in endovascular reperfusion therapy for medium vessel occlusion. Preprocedural angiogram showing left anterior cerebral artery A2 occlusion (arrow) (A). Postprocedural angiogram showing recanalization with residual stenosis (arrows) (B and C).

**Fig. 3:**
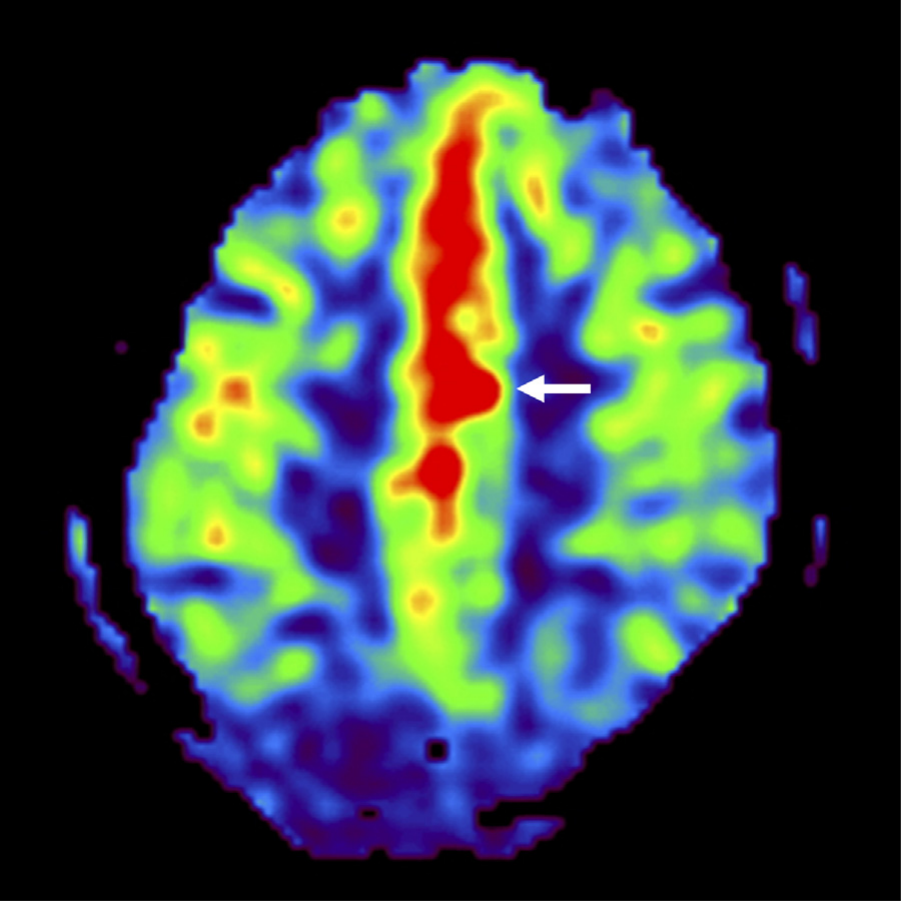
Postoperative arterial spin labeling demonstrating hyperperfusion in the left anterior cerebral artery area (arrow).

A 70-year-old woman with a history of hypertension and dyslipidemia experienced weakness in her right lower extremity upon waking and was unable to move. Two days earlier, she had also fallen because of lower extremity weakness. She was found by her family, immobile and with slurred speech, and was transported to our hospital by ambulance. On arrival, she had mild right lower extremity paralysis and a National Institutes of Health Stroke Scale (NIHSS) score of 1; however, her symptoms appeared better compared with symptom onset. Magnetic resonance imaging (MRI) revealed multiple fresh infarcts in the left frontal lobe. She was hospitalized urgently with AIS. However, her neurological symptoms worsened (NIHSS: 22) and she was referred to our department. MRI and MR angiography demonstrated poor delineation of the left ACA, with no apparent changes in the infarcts compared with the previous MRI findings (Fig. 1). Angiography revealed left ACA A2 occlusion (Fig. 2A); therefore, we performed EVT. Antegrade flow was obtained by mechanical disruption and intra-arterial thrombolysis. However, because of immediate reocclusion, we added mechanical disruption and intravenous infusion of ozagrel sodium (a thromboxane A2 synthase inhibitor), and finally achieved recanalization with residual stenosis (Figs. 2B and C). We diagnosed atherothrombotic brain infarction (ATBI) and administered aspirin, clopidogrel, rosuvastatin, argatroban (a direct thrombin inhibitor), and edaravone (a free radical scavenger) under blood pressure control. The day after the procedure, MRI/MR angiography showed no obvious infarct enlargement or intracranial hemorrhage and no reocclusion. However, the patient had prolonged consciousness disturbance, severe hemiparesis, and aphasia (NIHSS: 8). We considered CHS and continued treatment under strict blood pressure control. On postoperative day 2, arterial spin labeling (ASL) confirmed hyperperfusion in the left ACA area (Fig. 3). The symptoms gradually improved (NIHSS: 1, 1 week postoperatively), and she was discharged home. During the 2-year follow-up, no recurrence was observed.

## Discussion

Herein, we presented a rare case of CHS after successful EVT for acute primary MeVO (ACA A2 segment). CHS is a rare but serious complication after revascularization procedures for cerebrovascular diseases [1]. CHS can develop after AIS treatment, including intravenous thrombolysis and EVT for LVO [[1], [2], [3],^5^]. A previous study found that approximately half (48%) of the patients developed hyperperfusion after successful EVT for AIS with LVO, and that hyperperfusion affected the patients’ symptoms, hemorrhagic transformation, and outcomes [3]. However, to the best of our knowledge, there are no previous reports describing CHS after EVT for MeVO.

CHS remains unclear regarding its exact cause and pathogenesis. Although many mechanisms have been postulated to contribute to the development of hyperperfusion, impaired cerebral autoregulation has been recognized as the most important [1]. Reduced cerebral vascular reserve, considered a marker of cerebral autoregulation, is a reported risk factor for CHS [6]. Our patient developed CHS in spite of MeVO, and we considered the following 2 explanations: First, we considered the stroke etiology in our case to be ATBI, on the basis of the course of symptoms and the residual stenotic lesion after EVT (Figs. 2B and C). Chronic cerebral ischemia may have been caused by atherosclerotic ACA stenosis. Shimonaga et al. [3] reported that hyperperfusion tended to occur more frequently in large artery atherosclerosis (ie, ATBI) compared with cardioembolism. Additionally, our patient developed cerebral ischemic symptoms 2 days prior to admission and experienced stroke progression after hospitalization. This clinical course suggests that cerebral ischemia may have been at a critical level. Second, intraoperative recanalization, reocclusion, and recanalization occurred in this patient, which was expected to cause significant hemodynamic changes with repeated cerebral ischemia load.

In our case, ASL confirmed hyperperfusion, which was useful in understanding the pathophysiology and determining the treatment strategy (Fig. 3). Previous studies have reported the simplicity and usefulness of ASL for the detection of hyperperfusion [1,3,5]. Potential imaging findings of CHS on different MRI sequences have been reported to include patchy or diffuse white matter edema, focal infarction, and petechial hemorrhage [1]. Our patient had CHS in the left ACA area, especially in the supplementary motor cortex, which may have been difficult to identify on the basis of symptoms alone. However, ASL clearly demonstrated hyperperfusion, and these findings led to a diagnosis of CHS (Fig. 3). ASL was useful in detecting hyperperfusion in this case.

## Conclusion

We presented an extremely rare case of CHS after EVT for MeVO (ACA A2 segment) in a patient with ATBI. This report provides evidence that CHS can occur even after EVT for MeVO. ASL was useful in detecting hyperperfusion and contributed to the diagnosis of CHS.

## Patient consent

This study was approved by the institutional review board, and informed consent was obtained from the patient.

## Ethical statement

All procedures performed in studies involving human participants were in accordance with the ethical standards of the institutional and/or National Research Committee and with the 1964 Helsinki Declaration and its later amendments or comparable ethical standards. The study was approved by the Ethics Committee of Nakamura Memorial South Hospital (No. S2022122001).

## CRediT authorship contribution statement

**Hideki Endo:** Conceptualization, Methodology, Validation, Formal analysis, Investigation, Resources, Data curation, Writing – original draft, Writing – review & editing, Visualization, Project administration. **Kohei Ishikawa:** Validation, Formal analysis, Resources, Writing – review & editing. **Ryota Nomura:** Validation, Formal analysis, Resources, Writing – review & editing. **Daishi Yamaguchi:** Validation, Resources, Writing – review & editing. **Koichiro Shindo:** Resources, Writing – review & editing. **Koji Oka:** Validation, Resources, Supervision. **Hirohiko Nakamura:** Supervision.
